# An Unusual Condition

**Published:** 1910-07-15

**Authors:** L. P. Haskell

**Affiliations:** Chicago, Ills.


### AN UNUSUAL CONDITION.

BY DR. L. P. HASKELL, CHICAGO, ILLS.

I am often surprised at peculiar conditions found in the mouth, differing from anything before realized in a long and exclusive experience in the specialty of artificial dentures.

A lady came to me recently from Colorado, by the advice of a dentist. Fifteen years ago all her teeth had been extracted. She had worn with fair success an upper denture, but had been unable to wear a lower one. Why she was unable to secure a successful result within a year or two I can not understand, although several dentists had undertaken the case. But now, after the lapse of years, new conditions have arisen. It is usually considered that excessive disappearance of the ridge is owing to the wearing of a denture. In this case there is left a flat narrow ridge, with no possible chance for the plate to extend one iota on the lingual side. This, however, does not prevent a plate being worn with a fair degree of comfort.

This is not the worst feature of the case. The fact that there has been nothing on the lower jaw has resulted in a condition difficult to be overcome. The tongue has become accustomed to no interference in its movements, and I apprehend an enlargement. When the mouth opens, the tongue presses forward. Then the lower lip, having nothing to interfere, has pressed inward over the jaw, the muscles have contracted and a serious abnormal condition results.

Upon inserting a lower denture she complained that the tongue pushed the plate. Of course, this was to be expected. Later she complained that the lip pushed the plate inward—an inevitable result.

Another result is the change which has taken place in the coronoid process. Just what it is I can not tell, but the result is that while there is plenty of room for the anterior teeth in length, there is room only for two short molars on the upper jaw and one short molar on the lower jaw. When it is realized that three large molars were on both jaws, it is manifest that absorption has taken place in the coronoid process; at least it so appears to me.

One reason why a lower set could not be worn with the upper set she wore was in the position of the posterior teeth, the molars closing inside the lower ridge and being too long.

I told the patient it would require months for the tongue and lip to become accustomed to what is now an obstruction to both, before she could use the teeth to advantage, although she wore the plate with comfort otherwise, and restoration of features.

Will some dentist who is posted in the matter inform me if I am correct in my guess as to the change in the coronoid process?

